# Perspective matters: assessment of medical students' communication and interpersonal skills by simulated patients from the internal and external patient perspective

**DOI:** 10.3205/zma001478

**Published:** 2021-04-15

**Authors:** Sarah Prediger, Sigrid Harendza

**Affiliations:** 1 Universitätsklinikum Hamburg-Eppendorf, III. Medizinische Klinik, Hamburg, Germany

**Keywords:** assessment, communication, competence, interpersonal skills, patient perspective, simulated patients

## Abstract

**Background: **Communication and interpersonals skills are important qualities of professionalism in medicine. In medical curricula, they are usually acquired in communication trainings and assessed in OSCEs. Studies show correlations as well as differences between communication ratings of examiners and simulated patients. In our study, simulated patients assessed medical students’ communication and interpersonal skills after a consultation hour from the internal and the external patient perspective.

**Methods: **In December 2019, 52 final-year medical students participated in a consultation hour as part of a simulated first day of residency assessment. They were assessed twice with a questionnaire for communication and interpersonal skills (ComCare) by the simulated patients:

1. directly after each consultation from the internal perspective of the patient’s view (internal perspective) and
2. four month later from the 208 consultation videos from an external perspective of the patient’s view (external perspective).

All eight ComCare items were assessed on a five-point Likert scale (1=full disagreement to 5=full agreement). Differences between the item means of internal and external perspective were examined by paired t-tests.

**Results: **Overall, significantly higher ratings were found for all ComCare items from the external perspective except for the item “Interest”. Ratings for the items “Language” and “Listening” were significantly higher from the external perspective for all simulated patients. Significantly higher ratings for all items from the external perspective were observed for two simulated patients.

**Conclusion: **Simulated patients’ ratings after a conversation seem to represent a more authentic view on students’ communication and interpersonal skills because of the emotionally experienced situation. The evaluation of those skills from a simulated patient perspective could be a valuable complement to communication ratings by examiners.

## Zusammenfassung

**Hintergrund:** Kommunikative und zwischenmenschliche Fähigkeiten sind wichtige Eigenschaften der medizinischen Professionalität. In medizinischen Curricula werden sie üblicherweise in Kommunikationstrainings erworben und in OSCEs geprüft. Studien zeigen sowohl Korrelationen als auch Unterschiede zwischen den Kommunikationsbewertungen von Prüfer*innen und simulierten Patient*innen. In unserer Studie bewerteten simulierte Patient*innen die kommunikativen und interpersonellen Fähigkeiten von Medizinstudierenden nach einer Sprechstunde aus der internen und externen Patient*innenperspektive.

**Methoden: **Im Dezember 2019 nahmen 52 Medizinstudierende im Praktischen Jahr an einer Sprechstunde teil, die zum Training eines simulierten ersten Arbeitstages in der ärztlichen Weiterbildung gehörte. Sie wurden zweimal mit einem Fragebogen für kommunikative und interpersonelle Fähigkeiten (ComCare) von den simulierten Patient*innen bewertet:

1. direkt nach jeder Konsultation aus der internen Perspektive der Patient*innensicht (Innenperspektive) und
2. vier Monate später anhand der 208 Konsultationsvideos aus der externen Perspektive der Patient*innensicht (Außenperspektive).

Alle acht ComCare-Items wurden auf einer fünfstufigen Likert-Skala bewertet (1=volle Ablehnung bis 5=volle Zustimmung). Unterschiede zwischen den Item-Mittelwerten der Innen- und Außenperspektive wurden mittels gepaarter t-Tests untersucht.

**Ergebnisse: **Insgesamt wurden für alle ComCare-Items, außer für das Item „Interesse“, signifikant höhere Bewertungen aus der Außenperspektive gefunden. Die Bewertungen für die Items „Sprache“ und „Zuhören“ waren aus der Außenperspektive für alle simulierten Patient*innen signifikant höher. Signifikant höhere Bewertungen für alle Items aus der Außenperspektive wurden bei zwei simulierten Patient*innen beobachtet.

**Schlussfolgerungen: **Die Bewertungen von simulierten Patient*innen nach einem Konsultationsgespräch scheinen aufgrund der emotional erlebten Situation eine authentischere Sicht auf die kommunikativen und interpersonellen Fähigkeiten der Studierenden darzustellen. Die Bewertung dieser Fähigkeiten aus der Perspektive simulierter Patient*innen könnte eine wertvolle Ergänzung zu den Kommunikationsbewertungen durch Prüfer*innen darstellen.

## Introduction

Communication and interpersonals skills are important qualities of professionalism in medicine [1] and crucial elements in the delivery of good medical care and patient satisfaction [2], [3]. Undergraduate medical students usually receive communication trainings addressing empathy and interpersonal skills in their curriculum [4] and the assessment of communication skills often takes place in objective structured clinical examinations (OSCE) [5]. With respect to communication skills, mild, but significant correlations between simulated patients and examiners were found for warmth of greeting, listening skills, respect, and concern for the patient as a person in an OSCE [6]. While some studies found moderate to high correlations between simulated patients and examiners empathy ratings [7], [8], higher empathy ratings by simulated patients compared with examiner ratings in a clinical exam setting have been detected [9]. This study suggested further training for simulated patients to standardize empathy ratings and improve inter-rater reliability. Trained simulated patients have been shown to act as consistent raters for communications skills [10]. The research question for our study was, whether trained simulated patients assessed medical students’ communication and interpersonal skills after physician-patient encounters from the internal perspective of the patient’s view (short: internal perspective) in the same way than from an external perspective of the patient’s view (short: external perspective) when rating the videographed interviews four months later. We hypothesize that a rating from these two different perspectives will lead to different assessments.

## Project description and methods

Based on a validated 360-degree assessment for medical students, which simulates a first day of residency [11], we developed a competence-based training for final-year medical students in the newly founded Center for Training and Assessment of Medical Competences at the University Hospital Hamburg Eppendorf. In this training, participants complete three phases in the role of a physician:

1. a consultation hour with four simulated patients per participant,
2. a patient-management-phase with an online-patient-documentation-form, and
3. a case presentation of one of the simulated patients.

In December 2019, 52 medical students in the first third of their final year participated in this training. The eight simulated patients – detailed role descriptions are described elsewhere [12] filled out a questionnaire for communication and interpersonal skills (ComCare) after every consultation [13]. Its eight items were assessed on a five-point Likert scale (1=full disagreement to 5=full agreement). All consultations were videographed (N=208). All simulated patients had received a rater training in December 2019. They rehearsed their respective patient role with either a real physician or a final year student who acted as their interview partners. After each interview, they filled out the ComCare questionnaire. The ComCare items were discussed and typical anchors to recognize behaviour linked to each item were defined. 

In April 2020, four month after the assessment, the same eight simulated patients watched their own consultation videos and filled out the ComCare again. All simulated patients received a written briefing before they rated the videos. They were told to rate the participants from the patient perspective, focussing on communicative and interpersonal aspects and not on medical or other issues. The time distance of four month before the second rating was chosen to give the actors additional opportunity to have achieved emotional distance from their own role. The videos were rated by the actors in the same chronological order and in groups of four videos similar to the rating during the original 360-degree assessment.

Using SPSS Statistics 26 for statistical analysis, we calculated means and standard deviations for all ComCare items in total and per simulated patient. Additionally, we used paired t-tests to examine differences between both measurements (I: internal perspective, directly after each consultation, E: external perspective, four month later after watching their consultation videos). Due to multiple testing, p-Values were Bonferroni corrected. Cohen’s d was calculated for effect sizes. Additionally, we calculated the mean differences between both ratings (internal and external perspective) and conducted an ANOVA with those for the items “language” and “listening”, which showed significant differences for all simulated patients. For post-hoc testing, we used Bonferroni corrections. 

## Results

Overall, simulated patients assessed all ComCare items significantly higher from the external perspective except for the item “Showing sincere interest” (see table 1 (Tab. 1)). The greatest difference with large effect sizes between assessment from external versus internal perspective was found for the items “Language” (2.43 points, d=8.94) and “Listening” (1.68 points, d=3.97). Significantly higher ratings for these two items were also observed for all simulated patients individually from the external perspective (see table 2 (Tab. 2)). For all items, significantly higher ratings were only observed for SP3: 46-year-old man with severe abdominal pain (abdominal migraine) and SP4: 45-year-old woman with dizziness and malaise (second degree atrioventricular block Mobitz type II). We found significant differences between the mean differences of both ratings for “language” and “listening” in all simulated patients (see table 3 (Tab. 3)). The greatest difference for “language” was found for SP6 (a talkative and cooperative patient) and for “listening” for SP7 (a friendly, thankful patient with intense pain). SP8 (a patient with fear of cancer) showed the lowest mean differences between the two ratings for “language” and SP1 (a taciturn and suborn patient) for “listening”. 

## Discussion

Rating of medical students’ interpersonal skills or empathy in communication situations with simulated patients can be carried out in different ways: by only examiners directly after the consultation [14], [15], by the simulated patients themselves and by examiners directly after the consultation [6], [7], [8], [9], or by examiners watching video recorded communication situations at a convenient time [16], [17]. In our study, simulated patients rated the same consultations from the patient perspective directly after the consultation and four months later from the external perspective of the patient’s view, watching their videos. Interestingly, all simulated patients gave much better scores for “Use of plain language” and “Attentive listening” from the external perspective than from the internal, emotionally altered patient perspective. Real patients reported that they sometimes felt that doctor was not listening or used words they did not understand [18], [19]. Our simulated patients’ lower ratings from the internal perspective could be a result of their perceived impression whereas they might have rated the video with emotional distance from the external perspective. Two simulated patients who played roles with very debilitating emotional states (SP3 is very angry and SP4 is stressed in her job), rated all aspects of the ComCare lower from the internal than from the external perspective. Regarding the aspect of “listening”, the mean difference between the two ratings was, for example, significantly higher for a patient with intense pain (SP7) compared to a taciturn, stubborn patient (SP1).This gives another clue, that severe emotional alteration (e.g. by pain) can greatly hamper a (simulated) patient’s evaluation of a (simulated) physician’s communication skill. It has been shown before that ratings of communication skills are not independent of case content [20] and the acted emotions might have influenced the rating from the internal perspective in our setting versus the more distanced rating from the external perspective without the emotions of patient role. 

A strength of this study is that we deployed only one actor per patient role which supports high standardisation for role playing and rating. A limitation is that the actors could have gathered experiences between the two points of measurement, which could have influenced their rating at the second time point. Furthermore, we can only provide data from eight simulated patient roles, which limits the generalizability of our results. However, this pilot study provides first, interesting findings which can be explored further. 

## Conclusion

Simulated patient rating of communication and interpersonal skills after a conversation includes the internal patient perspective which is influenced by the patient’s emotions and therefore seems to represent a more authentic patient view. We conclude, that the evaluation of communication and interpersonal skills from the emotionally involved simulated patient perspective can be a valuable complement to communication ratings by examiners. Students could benefit from the additional learning experience that feedback from an emotionally involved simulated patient can differ from the feedback they receive from an external perspective, which is mostly based on professional aspects of communication.

## Funding

This work was supported by the Joachim Herz Stiftung, the Medical Faculty of Hamburg University, and the University Hospital Hamburg-Eppendorf.

## Acknowledgements

We thank all attending medical students for their participation. We are also very thankful to the actors and actresses Christian Bruhn, Christiane Filla, Franziska Herrmann, Ulrike Johannson, Thomas Klees, Thorsten Neelmeyer, Frank Thomé, and Claudia Wiedemer, who were very authentic patients and committed assessors during the SARS-CoV-2 panedmic. 

## Competing interests

The authors declare that they have no competing interests. 

## Figures and Tables

**Table 1:**
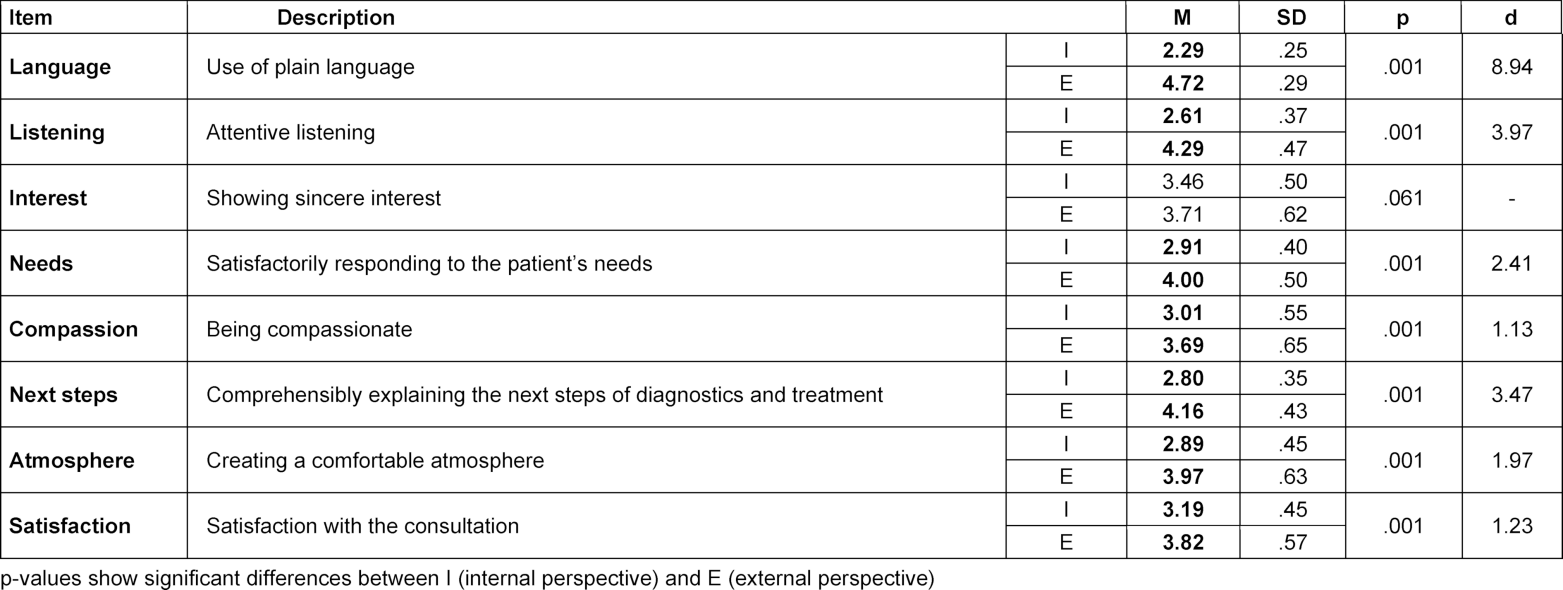
Means of ComCare items and results of paired t-test for all simulated patients in total

**Table 2:**
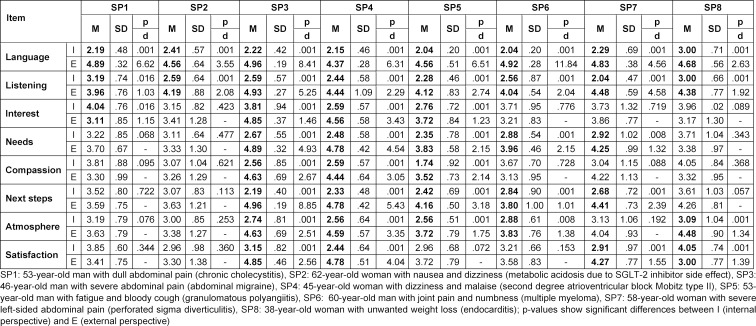
Means of ComCare items and results of paired t-test by simulated patients (SP)

**Table 3:**
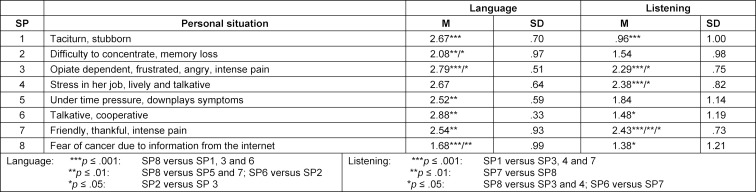
Mean differences between both ComCare ratings for the items “Language” and “Listening” by simulated patients (SP)

## References

[1] Bleakley A, Bligh J (2008). Students learning from patients: let's get real in medical education. Adv Health Sci Educ Theory Pract.

[2] Stewart MA (1995). Effective physician-patient communication and health outcomes: a review. CMAJ.

[3] Lloyd P, Lupton D, Donaldson D (1991). Consumerism in the health care setting: an exploratory study of factors underlying the selection and evaluation of primary medical services. Aust J Public Health.

[4] Sanson-Fisher R, Hobden B, Waller A, Dodd N, Boyd L (2018). Methodological quality of teaching communication skills to undergraduate medical students: a mapping review. BMC Med Educ.

[5] Guiton G, Hodgson CS, Delandshere G, Wilkerson L (2004). Communication skills in standardized-patient assessment of final-year medical students: a psychometric study. Adv Health Sci Educ Theory Pract.

[6] Greco M, Spike N, Powell R, Brownlea A (2002). Assessing communication skills of GP registrars: a comparison of patient and GP examiner ratings. Med Educ.

[7] Wright B, McKendree J, Morgan L, Allgar VA, Brown A (2014). Examiner and simulated patient ratings of empathy in medical student final year clinical examination: are they useful?. BMC Med Educ.

[8] O'Connor K, King R, Malone KM, Guerandel A (2014). Clinical examiners, simulated patients, and student self-assessed empathy in medical students during a psychiatry objective structured clinical examination. Acad Psychiatry.

[9] Chen JY, Chin WY, Tsang JP (2020). How clinician examiners compare with simulated patients in assessing medical student empathy in a clinical exam setting. Med Teach.

[10] Mafinejad MK, Rastegarpanah M, Moosavi F, Shirazi M (2017). Training and validation of standardized patients for assessing communication and counseling skills of pharmacy students: a pilot study. J Res Pharm Pract.

[11] Prediger S, Schick K, Fincke F, Fürstenberg S, Oubaid V, Kadmon M, Berberat PO, Harendza S (2020). Validation of a competence-based assessment of medical students' performance in the physician's role. BMC Med Educ.

[12] Harendza S, Gärtner J, Zelesniack E, Prediger S (2020). Evaluation of a telemedicine-based training for final year students including simulated patient consultations, documentation, and case presentation. GMS J Med Educ.

[13] Gärtner J, Prediger S, Harendza S (2021). Development and pilot test of ComCare – a questionnaire for quick assessment of communicative and social competences in medical students after interviews with simulated patients. GMS J Med Educ.

[14] Scheffer S, Muehlinghaus I, Froehmel A, Ortwein H (2008). Assessing students' communication skills: validation of a global rating. Adv Health Sci Educ Theory Pract.

[15] Monti M, Klöckner-Cronauer C, Hautz SC, Schnabel KP, Breckwoldt J, Junod-Perron N, Feller S, Bonvin R, Huwendiek S (2020). Improving the assessment of communication competencies in a national licensing OSCE: lessons learned from an experts' symposium. BMC Med Educ.

[16] Vogel D, Meyer M, Harendza S (2018). Verbal and non-verbal communication skills including empathy during history taking of undergraduate medical students. BMC Med Educ.

[17] Kiehl C, Simmenroth-Nayda A, Goerlich Y, Entwistle A, Schiekirka S, Ghadimi BM, Raupach T, Koenig S (2014). Standardized and quality-assured video-recorded examination in undergraduate education: informed consent prior to surgery. J Surg Res.

[18] Adamson TE, Gullion DS (1984). Physician-patient communication and medical malpractice. Mobius.

[19] Beck RS, Daughtridge R, Sloane PD (2002). Physician-patient communication in the primary care office: a systematic review. J Am Board Fam Pract.

[20] Zoppi K, Epstein RM (2002). Is communication a skill? Communication behaviors and being in relation. Fam Med.

